# Tuning the Crystallite Size, Shape, and Magnetic Properties of Fe_3_O_4_ Nanoparticles Using Annealing

**DOI:** 10.3390/ma19132911

**Published:** 2026-07-07

**Authors:** Riddhiman Medhi, Arati G. Kolhatkar, Yi-Ting Chen, Mohammad Khodadadi, Nhat Ngo, Rohan Dhall, Jacob Magdon, Pailinrut Chinwangso, Alba M. Valero, Francisco C. Robles Hernandez, Dimitri Litvinov, T. Randall Lee

**Affiliations:** 1 Department of Chemistry, University of Scranton, 800 Linden Street, Scranton, PA 18510, USA; 2 Department of Chemistry and the Texas Center for Superconductivity, University of Houston, Houston, TX 77204, USA; 3 Department of Electrical and Computer Engineering, University of Houston, Houston, TX 77204, USA; 4 National Center for Electron Microscopy, Molecular Foundry, Lawrence Berkeley National Laboratory, Berkeley, CA 94720, USA; 5 Department of Engineering Technology, University of Houston, Houston, TX 77204, USA; 6 Advanced Manufacturing Institute, University of Houston, Houston, TX 77204, USA

**Keywords:** Fe_3_O_4_, nanocubes, nanospheres, crystallinity, annealing, magnetization, shape transition

## Abstract

This study examines the effect of annealing on 135 nm Fe_3_O_4_ nanospheres and establishes a direct correlation among particle shape, crystallite size, and magnetic properties. Polycrystalline nanospheres and highly crystalline nanocubes with an equivalent diameter/body diagonal of 135 nm were synthesized via solvothermal and thermal decomposition methods, respectively. Scanning electron microscopy (SEM) revealed that the nanospheres developed smoother surfaces and gradually transformed toward a cubic morphology upon annealing, with increasing temperature and duration. Vibrating sample magnetometry (VSM) measurements showed that both saturation magnetization and coercivity increased with annealing as the particles evolved toward cube-like morphology and larger crystallite size, indicating that the magnetic properties of Fe_3_O_4_ nanoparticles are strongly dependent on crystallite size and shape. Nanospheres annealed between 500 and 850 °C exhibited increases in both crystallite size and saturation magnetization; however, coercivity decreased at 850 °C, where the crystallite size was maximal. Annealing at 700 °C for 12 h resulted in enhanced crystallite size and improved magnetic properties. Prolonged annealing at 700 °C (24 h) yielded the largest crystallite size but led to a significant reduction in saturation magnetization. This study demonstrates a clear correlation between magnetic properties, crystallinity, and morphology in nanoparticles beyond the superparamagnetic size regime (e.g., 135 nm). It further provides a strategy for tuning structural parameters that govern magnetic behavior and establishes an alternative, more facile route to obtain Fe_3_O_4_ nanospheres with crystallite sizes and magnetic properties comparable to nanocubes obtained via direct synthesis.

## 1. Introduction

Magnetic nanoparticles continue to be the focus of extensive research due to their applicability across a wide range of fields [1,2,3,4,5]. Among these materials, Fe_3_O_4_ nanoparticles are of particular interest because of their tunable physicochemical properties and broad applications, unlocking new innovations in medical diagnostics, cancer therapy, and drug delivery [1,2,3,4,5]. The influence of particle shape and the optimization of magnetic properties as a function of shape continue to garner attraction [6,7]. In an earlier work, Guardia et al. had studied the effects of reducing agent and surfactant on the shape and magnetic properties of Fe_3_O_4_ nanoparticles [8]. In sensing applications, our previous work demonstrated that cubic Fe_3_O_4_ nanoparticles with higher crystallinity exhibit higher saturation magnetization than spherical, polycrystalline counterparts. Specifically, Fe_3_O_4_ nanocubes outperformed nanospheres when compared on the basis of equivalent diameter/body diagonal (100–225 nm) or equal volume [9]. Using in-house sensing techniques, including giant magnetoresistance (GMR) and force-induced remanent magnetization spectroscopy (FIRMS), we further showed that the larger contact area of cubic magnetic nanoparticles (MNPs) enables stronger binding to sensing platforms and improved sensitivity. Despite these findings, a systematic understanding of the combined effects of shape and crystallite size on magnetic properties remains incomplete, particularly when comparing particles with similar crystallite sizes. This can be addressed either by synthesizing highly crystalline nanospheres with enhanced saturation magnetization (*M_s_*) or by increasing the crystallite size of as-synthesized polycrystalline nanospheres through thermal treatment. However, efforts to directly synthesize highly crystalline nanospheres in the size regime above 80 nm have been largely unsuccessful. Accordingly, this study focuses on enhancing the crystallite size of polycrystalline nanospheres via annealing and comparing their magnetic properties with those of nanocubes having equivalent diameter/body diagonal. The relationship among particle shape, crystallite size, and magnetic properties is examined to evaluate whether annealing can enhance saturation magnetization.

Several studies have investigated the effect of annealing on Fe_3_O_4_ nanoparticles. Yang et al. annealed Fe_3_O_4_ nanoparticles (7, 18, 42, and 53 nm) in the temperature range of 500−800 °C under an atmosphere of 5% O_2_ and 95% N_2_ [10]. This resulted in oxidation to α-Fe_2_O_3_ with increased coercivity (~6 kOe), accompanied by a transformation of cubic Fe_3_O_4_ into spherical and rhombohedral α-Fe_2_O_3_ [10]. In another study, Fe_3_O_4_ nanoparticles (10–40 nm) annealed at 50–500 °C retained superparamagnetic behavior across all temperatures. Details regarding the annealing medium were not reported. The *M_s_* of the as-prepared sample was 65.4 emu g^−1^ and increased to a maximum of 76.8 emu g^−1^ at 300 °C, followed by a decrease at higher annealing temperatures [11]. The Gacoin group employed post-synthesis heat treatment of 8 ± 2 nm γ-Fe_2_O_3_ nanoparticles to enhance magnetic anisotropy. The size distribution was preserved, and transformation to α-Fe_2_O_3_ was prevented by embedding the nanoparticles in a silica matrix. Increased anisotropy was observed after annealing at 100, 200, 300, 400, 500, and 600 °C in air, as well as upon doping with Co(II) ions [12]. Dar et al. reported the transformation of amorphous iron oxide (<5 nm and 60 nm) into monocrystalline Fe_3_O_4_ and Fe_2_O_3_ (α and γ phases) through annealing under N_2_ and ambient conditions, respectively [13]. Annealing at 200, 300, and 600 °C yielded monocrystalline Fe_3_O_4_, γ-Fe_2_O_3_, and α-Fe_2_O_3_ nanoparticles with high saturation magnetization and coercivity. Jafari et al. calcined Fe_3_O_4_ nanoparticles at temperatures ranging from 50 to 850 °C for 1 h in air and observed an increase in average crystallite size from 7.2 to 35.8 nm [14]. X-ray diffraction (XRD) analysis indicated that superparamagnetic Fe_3_O_4_ (7.2 nm crystallite size) transformed to ferromagnetic α-Fe_2_O_3_ in the range of 550–650 °C and to γ-Fe_2_O_3_ in the range of 750–850 °C.

Kalska-Szostko et al. varied synthetic methods to produce Fe_3_O_4_ nanoparticles with different surface chemistries and subsequently examined the effect of annealing over the temperature range of 50–500 °C using an air-flow furnace [15]. Regardless of the synthesis route, heating resulted in oxidation of magnetite to maghemite and hematite. Etemadifar et al. modified the surface chemistry by employing zucchini and pomegranate peel extracts rich in phenolic and hydroxyl groups [16]. After annealing at 550 °C for 2 h in air, *M_s_* increased from 21.4 to 45.8 emu g^−1^ relative to the as-synthesized nanoparticles. In another study involving thermal treatment of 13 ± 2 nm Fe_3_O_4_ nanoparticles, annealing at 950 °C resulted in the formation of hematite and either pure α-Fe or a mixture of α-Fe and olivine under oxygen and argon atmospheres, respectively [17]. Additionally, silica-coated Fe_3_O_4_ nanoparticles annealed at 950 °C in oxygen yielded ε-Fe_2_O_3_. Thermal annealing of Fe_3_O_4_ nanoparticles over the temperature range of 100–800 °C in the presence of oxygen led to the formation of maghemite between 200 and 400 °C and hematite above 500 °C. This study identified the transition temperatures and demonstrated an increase in crystallite size with increasing annealing temperature [18].

A more recent study examined the structural and magnetic properties of Fe_3_O_4_ after annealing at 250, 450, 650, and 850 °C for 2 h [19]. The authors reported morphological evolution from spherical to irregular shapes, an increase in crystallite size, and phase transformation to maghemite at 250 °C and hematite at 850 °C.

For applications such as sensing, nanoparticles with sizes beyond the superparamagnetic regime are often preferred due to their larger surface area for functionalization and higher saturation magnetization. However, the limited data available for this size regime highlights the need for systematic studies evaluating the effects of annealing temperature on morphology and crystallite size to obtain Fe_3_O_4_ nanoparticles with enhanced magnetic properties. Morphology is a critical factor influencing nanoparticle crystallinity, and the relationship between these parameters remains insufficiently understood. Accordingly, the present work focuses on synthesizing 135 nm polycrystalline, spherical Fe_3_O_4_ nanospheres first, and then annealing them at temperatures between 500 and 850 °C for durations of 1–12 h under oxygen-free conditions, with the aim of correlating changes in morphology, crystallite size, and magnetic properties while approaching cube-like crystallinity and magnetic properties.

## 2. Experimental Section

### 2.1. Materials

Iron(III) acetylacetonate (Fe(acac)_3_), iron(III) chloride hexahydrate [FeCl_3_·6H_2_O], sodium acetate, oleic acid, benzyl ether, ethylene glycol, and poly(vinylpyrrolidone) (PVP) were purchased from Sigma-Aldrich (St. Louis, MO, USA) and used as received. Water was purified to a resistivity of 18 MΩ·cm using a Milli-Q Academic Water Purification System (Millipore Corporation, Burlington, MA, USA).

All glassware and reaction vessels were cleaned with freshly prepared aqua regia solution (3:1 HCl/HNO_3_), rinsed thoroughly with deionized water and acetone, and dried in an oven prior to use.

### 2.2. Characterization Methods

Scanning electron microscopy (SEM) images were acquired using a Carl Zeiss LEO 1525 microscope (Carl Zeiss SMT AG, Oberkochen, Germany) operated at an accelerating voltage of 15 kV. Samples for SEM were prepared by depositing nanoparticle dispersions onto silicon wafers that had been cleaned with water and ethanol.

Transmission electron microscopy (TEM) and selected-area electron diffraction (SAED) were performed using a JEOL Ltd. JEM-2100 operated at 200 kV (JEOL Ltd., Akishima, Japan). High-angle annular dark-field scanning transmission electron microscopy (HAADF-STEM) imaging and energy-dispersive X-ray spectroscopy (EDS) were conducted using a Thermo Fisher Scientific Spectra 300 operated at 300 kV (Thermo Fisher Scientific, Waltham, MA, USA). TEM samples were prepared by depositing dispersions onto 300-mesh holey carbon-coated copper grids and drying overnight (Quantifoil Micro Tools GmbH, Großlöbichau, Germany).

Powder X-ray diffraction (XRD) patterns were collected using a Malvern Panalytical X’Pert PRO diffractometer with Cu Kα radiation (λ = 1.5406 Å) (Malvern Panalytical B.V., Almelo, The Netherlands). Concentrated nanoparticle suspensions in water/ethanol were drop-cast onto piranha-cleaned glass slides. Diffraction data were collected over the 2θ range of 10–100° with a scan time of 15 min. The Scherrer equation was used to estimate the crystallite size of the phases present in the nanoparticles [20].

Magnetic measurements, including saturation magnetization (*M_s_*), remanent magnetization (*M_r_*), and coercivity (*H_c_*), were performed using a Lake Shore Cryotronics vibrating sample magnetometer (VSM) 7300 Series (with LakeShore 735 Controller and LakeShore 450 Gaussmeter, Lake Shore Cryotronics, Inc., Westerville, OH, USA; Software Version 3.8.0).

Thermogravimetric analysis (TGA) was carried out using a TA Instruments TGA-2050 under a constant argon flow of 100 mL min^−1^. Samples were heated from 25 to 800 °C at 5 °C min^−1^ (TA Instruments, Inc., New Castle, DE, USA). The mass measurements were recorded with an accuracy of less than ±0.3%.

In situ TEM experiments were performed at Lawrence Berkeley National Laboratory (Berkeley, CA, USA) using a Thermo Fisher Scientific ThermIS equipped with an image corrector and operated at 300 kV (Thermo Fisher Scientific, Waltham, MA, USA). High-speed videos were recorded using a Ceta camera (Thermo Fisher Scientific, Waltham, MA, USA), and drift correction was performed with AXON software (Software Version 10.8.7). Samples were mounted on a Protochips heating and biasing holder.

### 2.3. Synthesis

**Synthesis of Fe_3_O_4_ Nanospheres.** Fe_3_O_4_ nanospheres with average diameters of approximately 135 nm were synthesized using a modified procedure based on the method reported by Deng et al. [9,21]. Iron(III) chloride hexahydrate (1.4 g) was dissolved in 15 mL of ethylene glycol, followed by the addition of sodium acetate (3.6 g). Upon addition of sodium acetate, the solution changed from orange to brown. After stirring for 30 min, the solution was rapidly transferred to a vigorously stirred solution of PVP (0.40 g) in 35 mL of ethylene glycol maintained at 180 °C. The mixture was stirred at 180 °C for 4–24 h, during which a black precipitate formed. The product was isolated by repeated washing with ethanol and Milli-Q water and dried under vacuum at room temperature.

**Synthesis of Fe_3_O_4_ Nanocubes.** Fe_3_O_4_ nanocubes with body-diagonal lengths of approximately 135 nm (edge length ≈ 80 nm) were synthesized using a previously reported method [9]. Iron(III) acetylacetonate and oleic acid were heated to 290 °C in benzyl ether under magnetic stirring. After 30 min, a black precipitate was obtained, washed repeatedly with ethanol, and dried under vacuum at room temperature. By varying the reactant concentrations and reaction time, nanocubes with tunable sizes were obtained.

### 2.4. Annealing Studies

Fe_3_O_4_ nanoparticles were annealed under high-purity argon (99.999%) using an MTI OTF-1200X Tube Furnace (MTI Corporation, Richmond, CA, USA). For each experiment, 3–7 mg of nanoparticles were placed in a quartz boat and inserted into the furnace tube. The tube was purged with argon for 1 min and then subjected to five pressurization–evacuation cycles, during which it was filled with argon to 2.5 bar and subsequently evacuated. During annealing, the argon pressure was maintained at 1.5 bar to minimize air ingress. All annealing experiments were conducted by heating from room temperature (20 °C) to the target temperature at a rate of 5 °C min^−1^. After reaching the desired temperature, samples were held for the specified time and then allowed to cool to room temperature under argon.

## 3. Results and Discussion

In our previous study, we noted that spheres and cubes with equivalent body diagonals and the same volume exhibited different magnetic characteristics and sensing capabilities [9]. This disparity in magnetic properties was also reflected in their crystallite sizes. To determine whether the superior magnetic properties of the cubes were a function of their higher crystallinity or their morphology, annealing experiments were conducted using nanospheres. For this study, 135 nm Fe_3_O_4_ nanospheres (NSs) were synthesized using previously reported procedures [9]. The nanospheres were annealed at temperatures ranging from 500 to 850 °C to examine changes in their crystalline structure. Preliminary annealing experiments in air promoted partial or complete oxidation to the non-magnetic Fe_2_O_3_ phase, resulting in a substantial decrease in the saturation magnetization of the nanoparticles. Therefore, all subsequent annealing experiments reported in this study were conducted under argon.

**Figure 1:**
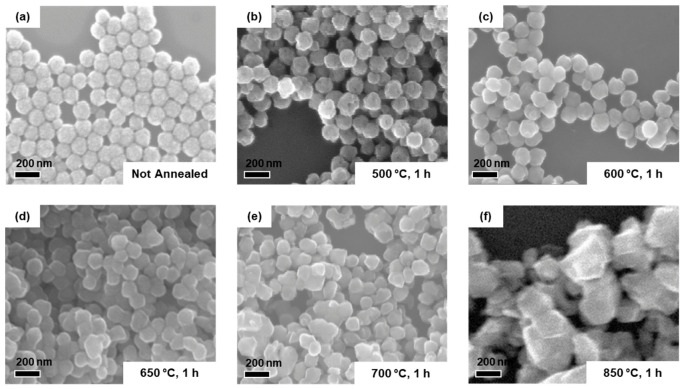
SEM images of (**a**) 135 nm Fe_3_O_4_ MNPs and samples annealed for 1 h at (**b**) 500 °C, (**c**) 600 °C, (**d**) 650 °C, (**e**) 700 °C, and (**f**) 850 °C.

The nanoparticles were imaged before and after annealing using scanning electron microscopy (SEM) to monitor morphological changes. Figure 1 shows SEM images of Fe_3_O_4_ NSs after annealing at 500, 600, 650, 700, and 850 °C for 1 h. As the annealing temperature increased, the MNPs exhibited progressively smoother surfaces and a change in shape. The nanospheres gradually transformed into cubic structures with increasing annealing temperature. Based on the SEM images, the morphology obtained after annealing at 700 °C for 1 h is closest to a cubic shape. However, at 850 °C, the annealing process led to complete aggregation of the nanoparticles.

**Figure 2:**
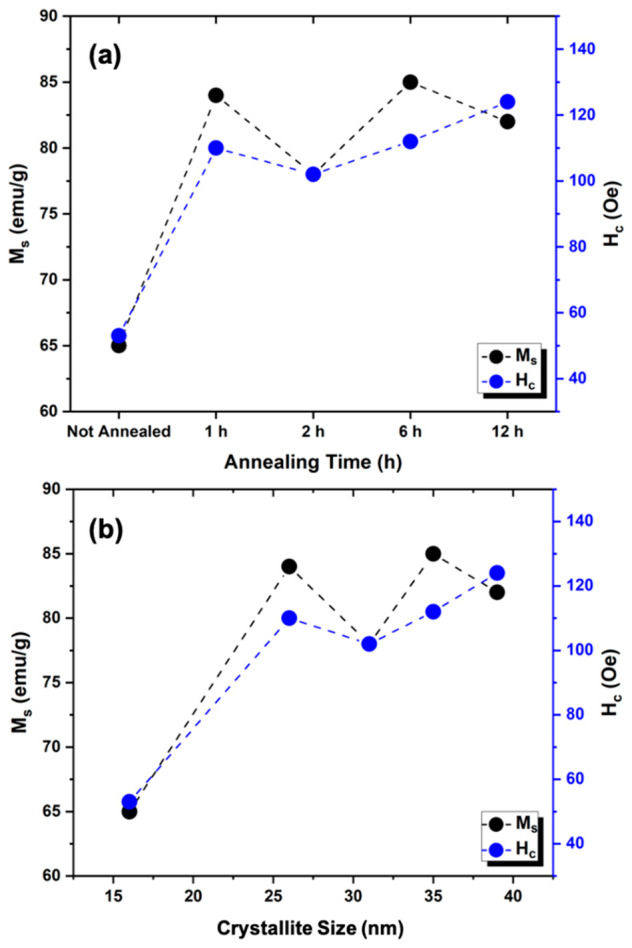
Effect of (**a**) annealing time and (**b**) crystallite size on the saturation magnetization (*M_s_*) and coercivity (*H_c_*) of 135 nm Fe_3_O_4_ MNPs and samples annealed for 1 h at 500, 600, 650, 700, and 850 °C.

To assess changes in crystalline structure, all nanoparticles were characterized using powder XRD before and after annealing. Magnetic properties were measured using a vibrating sample magnetometer (VSM). The crystallite size and magnetic properties of the MNPs annealed at the above temperatures are summarized in Table S1. The nanoparticles became more crystalline upon annealing (see Table S1 and Figure 2, Figure S1 and Figure S2), as indicated by an increase in crystallite size. Minimal changes were observed at 500 °C for 1 h, whereas more significant increases in crystallite size occurred at temperatures of 600 °C and above. The *M_s_* and coercivity (*H_c_*) values exhibited similar trends; however, a noticeable increase in these magnetic properties was already observed at 500 °C. The highest saturation magnetization (84 emu g^−1^) was observed for particles annealed at 700 °C for 1 h, while the highest coercivity (121 Oe) was observed for particles annealed at 650 °C. The largest crystallite size was obtained for particles annealed at 850 °C.

The 135 nm diameter of the nanospheres used in this study is slightly greater than the theoretical critical size of 76 nm reported for nanospheres. The experimentally determined critical size for cubic Fe_3_O_4_ nanoparticles is 160 nm, while the theoretically estimated value is 128 nm for Fe_3_O_4_ nanospheres [22,23,24]. The critical size corresponds to the transition between single- and multi-domain regimes and is typically correlated with particle size. However, magnetic behavior remains complex, and estimation of critical size based on correlations between magnetic properties and crystal structure continues to garner attention [23]. The linear dependence of *M_s_* and the correlation of *H_c_* (including the presence of a maximum) with nanoparticle size are well documented [23,25,26]. A similar trend is observed in this study; however, the focus here is on the crystallite size of the nanoparticles (Figure 2). After annealing at 650–700 °C, corresponding to a crystallite size of approximately 26–28 nm, the coercivity decreases, suggesting a transition to a non-coherent magnetization reversal mode [27].

**Figure 3:**
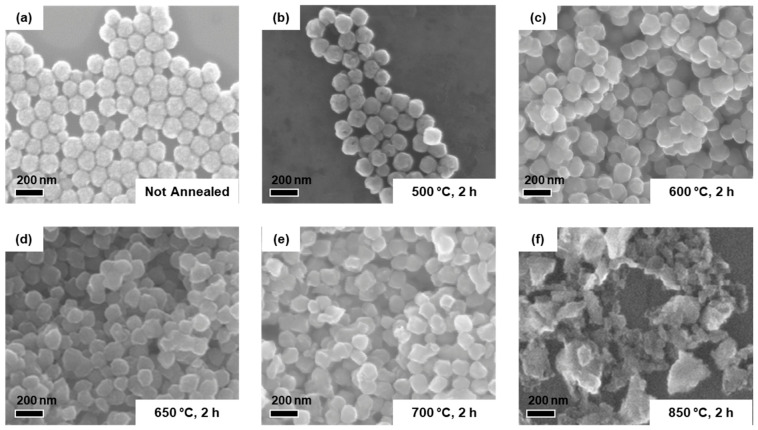
SEM images of (**a**) 135 nm Fe_3_O_4_ MNPs and samples annealed for 2 h at (**b**) 500 °C, (**c**) 600 °C, (**d**) 650 °C, (**e**) 700 °C, and (**f**) 850 °C.

**Figure 4:**
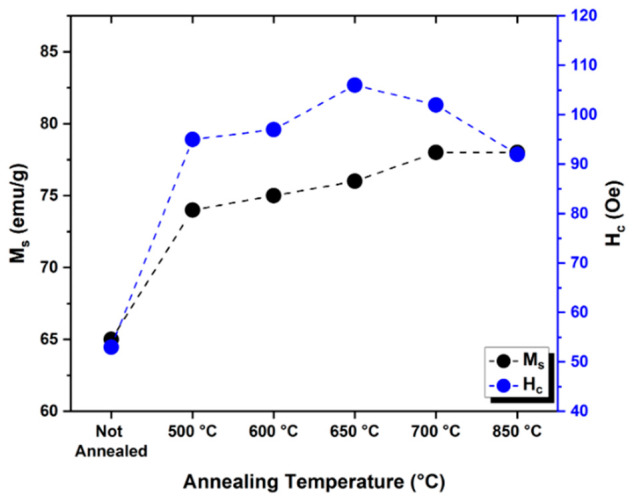
*M_s_* and *H_c_* of 135 nm Fe_3_O_4_ MNPs and samples annealed for 2 h at 500, 600, 650, 700, and 850 °C.

To evaluate whether the trend observed for 1 h annealing is consistent and to examine the effect of annealing duration, the nanospheres were also annealed at these temperatures for 2 h. Similar results were obtained, as seen in Figure 3 and Figure 4. The nanospheres progressively developed smoother surfaces and transformed toward cubic morphology with increasing annealing temperature, ultimately aggregating at 850 °C. The increase in *M_s_* and *H_c_* values was more pronounced at 500 °C for 2 h compared to 1 h, indicating that annealing duration also influences the magnetic properties. Consistent with the 1 h results, the highest *M_s_* value was observed for spheres annealed at 700 °C, while the highest *H_c_* values were observed for spheres annealed at 650 °C. Thus, in both cases, spheres annealed at 700 °C exhibited the highest saturation magnetization while maintaining a discrete morphology. Accordingly, additional experiments were conducted at 700 °C with varying annealing durations (1–24 h).

**Figure 5:**
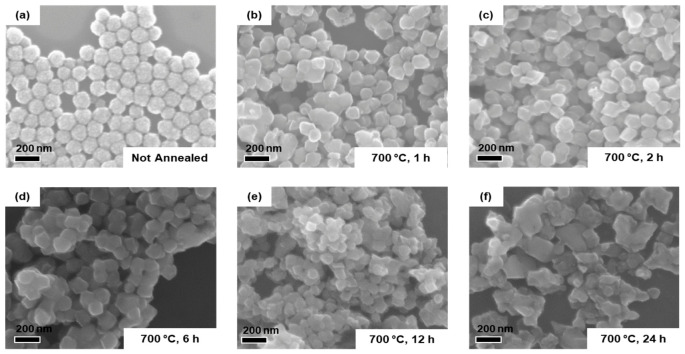
SEM images of 135 nm Fe_3_O_4_ MNPs: (**a**) as-synthesized particles and particles annealed at 700 °C for (**b**) 1 h, (**c**) 2 h, (**d**) 6 h, (**e**) 12 h, and (**f**) 24 h.

Figure 5 shows the morphology of nanospheres annealed at 700 °C for 1, 2, 6, 12, and 24 h. The particles gradually adopt a more cubic morphology as the annealing time increases. The XRD patterns in Figure S2 and the calculated crystallite sizes calculated from these data (Table S1) indicate that the particles also become more crystalline with increasing annealing time at 700 °C. However, after annealing for 24 h, the particles begin to turn orange and aggregate, resulting in a significant decrease in saturation magnetization. As shown in the XRD patterns in Figure S2, this sample contains an impurity phase corresponding to α-Fe_2_O_3,_ which leads to a loss of phase purity and a deterioration in magnetic properties. The α-Fe_2_O_3_ phase was observed only in this sample and was not seen in any of the other annealed samples. This suggests an argon atmosphere is sufficient to preserve the Fe_3_O_4_ phase during transformation up to 850 °C, unless subjected to annealing durations approaching 24 h.

**Figure 6:**
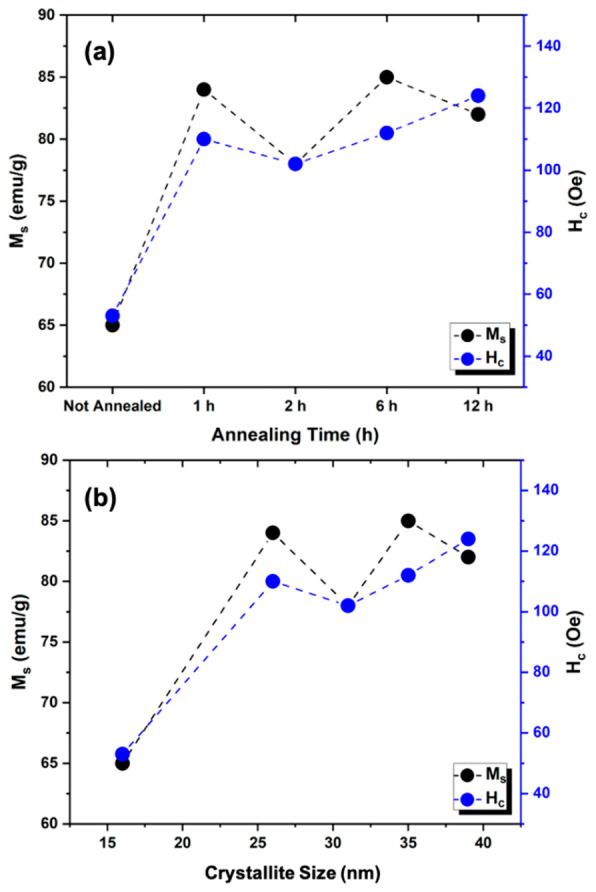
Effects of (**a**) annealing time and (**b**) crystallite size on the saturation magnetization (*M_s_*) and coercivity (*H_c_*) of 135 nm Fe_3_O_4_ MNPs annealed at 700 °C for 1, 2, 6, and 12 h. The sample annealed for 24 h exhibited oxidation to Fe_2_O_3_, as indicated by an orange color, and significant aggregation; therefore, its magnetic properties are not included in the plots.

Our previous study demonstrated that the saturation magnetization of nanocubes is higher than that of nanospheres with equivalent body diagonals (or diameters) and volumes [8]. Nanocubes obtained by direct synthesis exhibit an *M_s_* ratio (defined in Equation (1)) of approximately 1.3 relative to nanospheres with an equivalent body diagonal of 135 nm. Notably, the *M_s_* ratio of annealed nanospheres transformed into nanocubes relative to the corresponding unannealed nanospheres is also 1.3. This result indicates that the morphology transformation increases the crystallite size from 16 nm to 39 nm, and enhances the magnetic properties to a level comparable to that of directly synthesized, highly crystalline nanocubes (see Figure 6a,b).

(1) $$M_{s}\;ratio=\frac{{{(M}_{s})}_{nanocubes}}{{{(M}_{s})}_{nanospheres}}$$

where (*M_s_*)_nanocubes_ is the saturation magnetization of nanocubes (either as-synthesized or morphology-transformed), and (*M_s_*)_nanospheres_ is the saturation magnetization of nanospheres.

**Figure 7:**
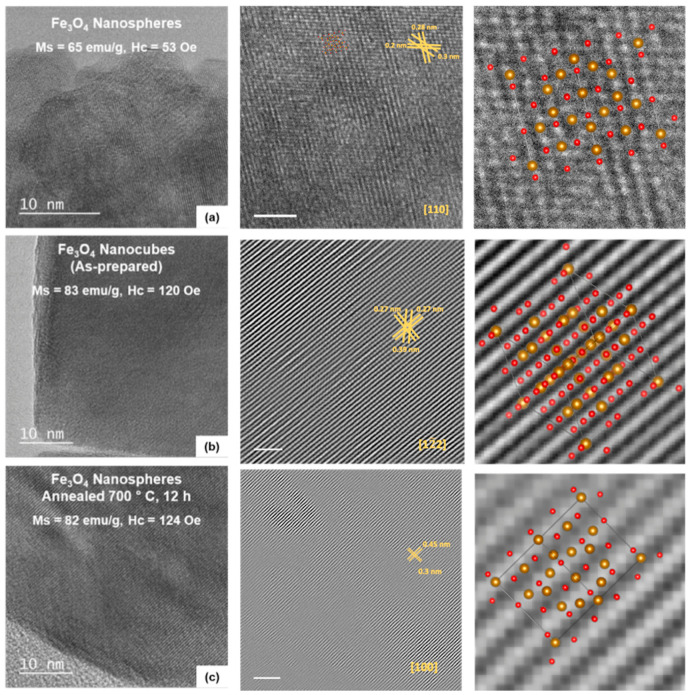
HRTEM images of (**a**) unannealed 135 nm Fe_3_O_4_ nanospheres, (**b**) unannealed 135 nm Fe_3_O_4_ nanocubes obtained by direct synthesis, and (**c**) 135 nm Fe_3_O_4_ nanospheres annealed at 700 °C for 12 h. Golden atoms represent Fe and red atoms represent O.

As shown in Figure 6a, annealing the nanospheres at 700 °C for 1, 2, 6, and 12 h results in a progressive increase in both saturation magnetization and coercivity, along with their transformation towards a cubic shape. After 12 h of annealing, the transformed particles exhibit magnetic properties comparable to those of nanocubes obtained by direct synthesis. Directly synthesized nanocubes exhibit a saturation magnetization of 83 emu g^−1^ and a coercivity of 120 Oe. These values are nearly identical to those of nanospheres annealed at 700 °C for 12 h, which exhibit a saturation magnetization of 82 emu g^−1^ and a coercivity of 124 Oe. High-resolution transmission electron microscopy (HRTEM) images in Figure 7 provide further insight into the evolution of the crystal structure. Importantly, the change in morphology to nanocubes upon slow, controlled annealing not only increases saturation magnetization and coercivity but also increases the flat contact surface area, which has been shown to be beneficial for sensing applications [9]. Nanospheres, on the other hand, generally cross cell membranes more readily than cubes; hence, this transformation to nanocubes, while increasing magnetic strength and surface area, could be accompanied by a loss of bioavailability [28].

The as-synthesized nanospheres are polycrystalline and consist of multiple smaller crystallites. The crystallite size of 16 nm, determined from XRD analysis, is consistent with the HRTEM observations. In contrast, nanocubes obtained by direct synthesis exhibit a highly ordered crystalline structure. Their crystallite size is 56 nm, which is substantially larger than that of nanospheres with an equivalent body diameter. Annealing the nanospheres at 700 °C for 12 h increases the crystallite size to 48 nm (Figure 6b), although this value remains lower than that of the directly synthesized nanocubes. Figure 7a,c show the HRTEM images of nanospheres before and after annealing at 700 °C for 12 h. In addition to adopting a morphology that approaches a cubic shape, as observed by SEM, the annealed nanospheres exhibit a crystal structure that closely resembles that of the directly synthesized nanocubes (Figure 7b,c).

The crystallinity index (*CI*) defined in Equation (2) provides a measure of the degree of crystallinity [8,29]. A lower *CI* indicates a lower degree of crystallinity.

(2) $$CI=\frac{particle\;size\;determined\;by\;SEM\;or\;TEM}{crystallite\;size}$$

As summarized in Table S1 and illustrated in Figure 1, the crystallite size increased as the annealing temperature was raised from 500 to 850 °C. A similar increase in crystallite size was observed when the annealing time at 700 °C was extended from 1 to 24 h (Table S1 and Figure 6b). The increase in CI with annealing temperature and duration indicates that annealing improves the crystallinity of the Fe_3_O_4_ nanoparticles. The selected-area electron diffraction (SAED) patterns shown in Figure S4 confirm the phase identity and indicate increased crystallinity with increasing annealing temperature, accompanied by a transformation from spherical to cubic morphology.

These results indicate that the magnetic properties of Fe_3_O_4_ MNPs are strongly correlated with crystallite size. In spherical particles, the constantly changing curvature of the surface limits the distance over which a crystallite can grow while maintaining a uniform crystallographic orientation. In contrast, cubic particles possess large planar facets that permit crystallites to extend over longer distances, resulting in larger coherent domains. Annealing reduces the density of broken bonds and decreases surface spin canting, which contributes to the increase in saturation magnetization [30]. Surface atoms suffer from broken translational symmetry with dangling bonds, varying super-exchange angles, and uncompensated “frozen” spin states [31,32]. As crystallite size increases, the volume fraction of this disordered surface layer decreases, leading to an increase in saturation magnetization [31,32]. Thermal annealing also heals lattice defects and helps recover interatomic bond lengths. Improved bond recovery in larger crystallites reduces cation inversion and strengthens the A—O—B super-exchange interactions, thereby increasing saturation magnetization [31,32,33,34]. The larger crystallite sizes observed after annealing are also evident in the HRTEM images. In addition to increasing crystallite size, annealing of the nanospheres induces a change in particle shape, making shape anisotropy an important contributor to the enhanced coercivity [22]. These observations are consistent with previous studies showing that larger crystallite sizes in Fe_3_O_4_ nanocubes lead to higher saturation magnetization due to reduced surface spin disorder [35,36,37]. Liu et al. reported that, for polycrystalline Fe_3_O_4_ nanospheres smaller than 250 nm, the saturation magnetization depends on both the particle diameter and the crystallite size [35].

Post-synthesis annealing has also been widely used to increase crystallite size and improve the magnetic properties of other ferrite nanoparticles, particularly in doped systems in which the incorporation of lower-moment cations reduces *M_s_* [38,39]. In inverse spinel ferrites with the general formula AB_2_O_4_, including Fe_3_O_4_ (which may be written as Fe^2+^Fe_2_^3+^O_4_), cations occupy tetrahedral (A) and octahedral (B) sites. In magnetite, Fe^2+^ ions occupy octahedral B sites, whereas Fe^3+^ ions are distributed equally between tetrahedral A and octahedral B sites [40]. The magnetic moments associated with the A and B sublattices are aligned antiparallel, and the net magnetic moment is determined by the difference between the two sublattice moments, which depends on both the number of unpaired electrons and the cation distribution [38,39,41].

**Figure 8:**
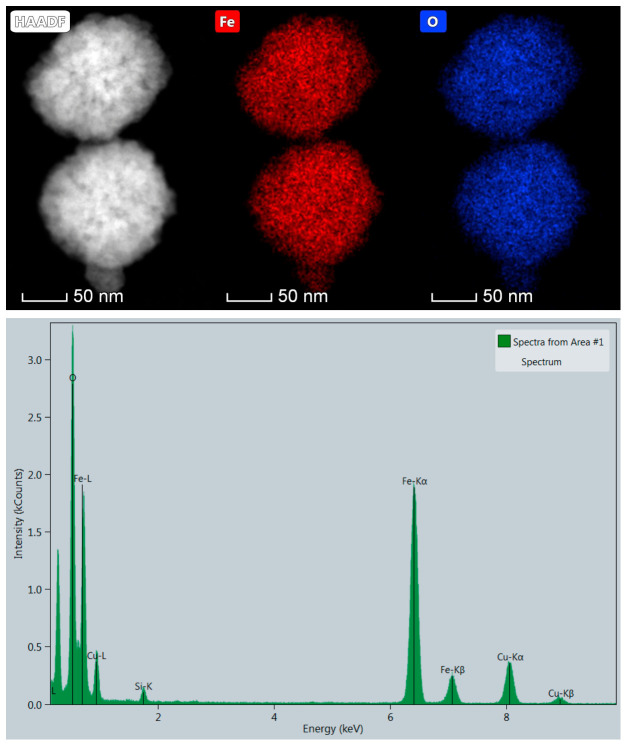
HAADF-STEM images and corresponding EDS elemental maps of the Fe_3_O_4_ nanospheres.

The increase in magnetization upon annealing may be attributed, in part, to cation redistribution, including a more ordered arrangement of Fe^2+^ ions in octahedral sites and changes in the Fe^3+^ occupancy of tetrahedral and octahedral sites [30,42,43,44]. X-ray magnetic circular dichroism (XMCD) studies by Ho et al. showed that polyhedral Fe_3_O_4_ nanoparticles exhibit comparable Fe^2+^ and Fe^3+^ occupancies at octahedral sites, whereas cubic particles display a higher Fe^3+^ occupancy at octahedral sites as a result of redistribution from tetrahedral sites [45]. Recent studies have shown the ability to study the cation occupancy and surface spins of ferrite nanoparticles using modified DFT methods such as DFT + U, DFT + U + V, and HSE06 [46,47,48,49]. Such structural modeling may be utilized to shed more light from a first-principles perspective on the relationship between morphology, crystallite size, and saturation magnetization. Liu et al. utilized a density functional tight binding with the Hubbard corrected (DFTB + U) method to study the atomic-scale structures of nanoparticles obtained by high-temperature annealing simulations of Fe_3_O_4_ nanospheres and nanocubes with a diameter of ~2.5 nm [46]. They concluded in their study that nanobues exhibit a higher saturation magnetization because the *N*(Fe_Oct_)/*N*(Fe_Tet_) in the nanocube (2.3) was higher than that of the nanosphere (2.0), after reconstruction of the particle through annealing and corresponding changes in the surface (001) facets. Cubic Fe_3_O_4_ nanoparticles also possess a larger fraction of atoms at planar surfaces, and surface layers terminated by octahedral sites are thermodynamically more stable than those terminated by tetrahedral sites. This surface stabilization likely drives the increased Fe^3+^ occupancy at octahedral sites in the nanocubes formed after annealing. Figure 8 presents high-angle annular dark-field scanning transmission electron microscopy (HAADF-STEM) images and corresponding energy-dispersive X-ray spectroscopy (EDS) elemental maps of the nanospheres. The maps demonstrate a homogeneous distribution of Fe and O throughout the particles. Signals from Cu, Si, and C originate from the TEM grid and support film.

**Figure 9:**
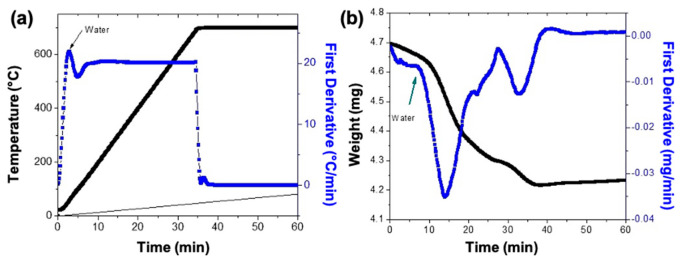
Thermogravimetric analysis of the Fe_3_O_4_ nanospheres; (**a**) heating profile and (**b**) mass loss curve. Note: the curves in blue and the respective “*Y*-axis also in blue” are the first derivatives for the heating and mass loss thermogravimetric curves respectively.

Thermogravimetric analysis (TGA) was performed on the as-synthesized nanospheres to monitor mass changes during heat treatment. The initial sample mass was 4.7 mg. As shown in Figure 9a, the sample was heated to 700 °C, which was reached after approximately 35 min. During the first 15 min, the sample lost adsorbed and residual water over the temperature range of approximately 75–135 °C. This process corresponded to a mass loss of 4.2 wt%, with a maximum rate of approximately 0.035 mg min^−1^. After dehydration, the sample continued to lose mass at a slower rate until reaching a final mass of approximately 4.2 mg, corresponding to a total mass loss of approximately 11 wt%. The thermogravimetric curve exhibits several regions of continuous mass loss with different rates, indicating that multiple processes occur during heating. Once the temperature reached 700 °C (after approximately 35 min), no further significant mass change was observed.

We noticed an additional mass loss of approximately 6.8 wt% after dehydration, which could be attributed primarily to oxygen release associated with structural rearrangement during the transformation from spherical to cubic morphology. This oxygen loss likely reflects local adjustment in stoichiometry and defect concentration that accompany particle reorganization rather than a bulk phase transformation. Consistent with this interpretation, the heating curve does not exhibit thermal events indicative of a first-order phase transition, and the heating rate remains essentially constant throughout the experiment. This observation is in agreement with the XRD results obtained after 1 h of annealing (Figure S1), which show no change in crystal phase.

The first derivative of the TGA curve was used to identify the temperatures and times at which the rates of mass loss were maximized, thereby providing insight into the stages at which the principal morphological changes occurred. A slight mass increase was observed after the sample reached 700 °C. This feature may arise from partial reoxidation or oxygen uptake as the structure approaches equilibrium and residual lattice strain is reduced at elevated temperatures.

This study provides multiple lines of evidence demonstrating that annealing of the nanospheres promotes crystallite growth and results in a substantial enhancement of the magnetic properties. Since annealing markedly increased both the crystallite size and the saturation magnetization of the nanospheres, we also investigated whether annealing nanocubes obtained by direct synthesis could further improve their magnetic performance. However, annealing the nanocubes at 700 °C for 2 h led to particle aggregation and deterioration of the magnetic properties (see Table S1). Similar behavior was observed for nanospheres annealed at 700 °C for longer than 12 h and for nanospheres annealed at 850 °C.

The decrease in saturation magnetization despite continued crystallite growth indicates that crystallite size alone does not determine the magnetic response. Preservation of a discrete nanoparticle morphology is also essential for optimizing magnetic properties. Because the cubic morphology already maximizes both the fraction planar surface facets and the occupancy of Fe^3+^ ions at octahedral sites, additional annealing does not improve the saturation magnetization (*M_s_*) or coercivity (*H_c_*). These results indicate that Fe_3_O_4_ nanoparticles with size in the 100 nm range are well beyond the superparamagnetic regime. Under the conditions examined here, the optimized magnetic properties, with *M_s_* ≈ 83 emu g^−1^ and *H_c_* ≈ 120 Oe, were obtained for particles exhibiting a cubic morphology. Further increases in *M_s_* and *H_c_* would likely require the direct synthesis of larger nanoparticles.

To visualize the morphological transformation, in situ TEM experiments were performed at the Lawrence Berkeley National Laboratory. Bulk observations were conducted, and a representative single-particle experiment is presented in Supplementary Video S1. In this experiment, an individual nanoparticle was monitored while being heated from room temperature to 800 °C. As depicted in Figure S5, hold times of 45 min at 700 °C and 75 min at 800 °C were used to approximate the annealing conditions employed in the ex situ experiments. The in situ TEM results directly confirm the annealing-induced transformation mechanism inferred from the ex situ characterization data. While there is some grain growth in the nanospheres beginning at 400 °C (see Table S1), morphology changes are only observed after 500 °C as seen in Supplementary Video S1. Therefore, the material can be mostly considered thermally stable below 500 °C. The nanoparticles remain discrete up to 700 °C, and chemically stable under argon even at 850 °C; however, prolonged annealing does compromise the chemical stability of the material, leading to the formation of the Fe_2_O_3_ phase after around 24 h of annealing (Figure S2).

In summary, annealing at 700 °C for 12 h provides a facile alternative route for obtaining highly crystalline and highly magnetic nanocubes in the ~100 size range. Additionally, time-controlled annealing can be leveraged to obtain quasi-cubic nanoparticles with crystallinity and magnetic properties that sit at any intermediate point within the nanosphere and nanocube spectra. The nanocube morphology obtained from controlled annealing of readily synthesized nanospheres provides advantages in self-assembly approaches on solid supports and for generating highly ordered Langmuir–Blodgett films, thereby enhancing fabrication capabilities for generating portable sensors and magnetic devices.

## 4. Conclusions

The crystallite size and magnetic properties of Fe_3_O_4_ nanospheres were significantly enhanced by post-synthesis annealing. As the annealing temperature and duration increased, the particle morphology gradually evolved from spherical to cubic. Annealing at 700 °C for 12 h produced nanocubes with the best combination of monodispersity, high crystallite size, and strong magnetic properties. Although nanospheres annealed at 700 °C for longer than 12 h exhibited larger crystallite sizes, their saturation magnetization decreased substantially. These results demonstrate that optimal magnetic performance requires both a large crystallite size and the retention of a discrete, uniform morphology.

Notably, the magnetic properties of nanospheres annealed at 700 °C for 12 h were essentially identical to those of nanocubes obtained by direct synthesis. The ratio of the saturation magnetization of the annealed, highly crystalline particles to that of the as-synthesized polycrystalline nanospheres was approximately 1.3. This value closely matches with the ratio observed between directly synthesized nanocubes and polycrystalline nanospheres, further supporting the conclusion that the enhanced magnetic properties arise primarily from improved crystallinity and the development of cubic morphology after annealing. Nanosphere synthesis typically utilizes inexpensive iron precursors, and simple solvents like water or alcohols, whereas direct nanocube synthesis requires expensive high-boiling-point organic solvents, specific capping agents, and higher synthesis temperature. Because the synthesis of Fe_3_O_4_ nanospheres is more straightforward and reproducible than the direct synthesis of nanocubes, controlled annealing of nanospheres offers a practical and cost-effective strategy for tuning magnetic properties and generating Fe_3_O_4_ nanocubes with optimized performance. For Fe_3_O_4_ nanoparticles in the ~100 nm size range, the highest values achieved in this study were a saturation magnetization of approximately 83 emu g^−1^ and a coercivity of approximately 120 Oe.

## Data Availability

The original contributions presented in this study are included in the article/Supplementary Material. Further inquiries can be directed to the corresponding author.

## Supplementary Materials

The following supporting information can be downloaded at: https://www.mdpi.com/article/10.3390/ma19132911/s1, Figure S1. X-ray diffraction (XRD) patterns of 135 nm Fe_3_O_4_ magnetic nanoparticles (MNPs) and samples annealed for 1 h at 500, 600, 650, 700, and 850 °C; Figure S2. XRD patterns of 135 nm Fe_3_O_4_ MNPs and samples annealed at 700 °C for 1, 2, 6, 12, and 24 h. Annealing at 700 °C led to oxidation, resulting in the formation of impurities associated with the α-Fe_2_O_3_ phase; Figure S3. (a) Saturation magnetization (*M_s_*) and (b) coercivity (*H_c_*) of 135 nm Fe_3_O_4_ MNPs and samples annealed at different temperatures for varying durations; Figure S4. Selected area electron diffraction (SAED) patterns of 135 nm Fe_3_O_4_ MNPs and samples annealed at 700 °C for 12 h; Figure S5. Heating curve for in situ transmission electron microscopy (TEM) measurements; Table S1. Magnetic properties of 135 nm Fe_3_O_4_ MNPs and samples annealed at different temperatures for varying durations. Video S1. Fe_3_O_4_ in-situ annealing.
