# Supplementary material for: Tuning the Crystallite Size, Shape, and Magnetic Properties of Fe3O4 Nanoparticles Using Annealing
Source: Materials (Basel). 2026 Jul 7;19(13):2911. doi: 10.3390/ma19132911 (PMC13362655; doi:10.3390/ma19132911)
Supplement: Supplementary file 1 [file materials-19-02911-s001.zip › SI - Effect of Annealing on Crystallinity, Shape, and Magnetic Properties_submitted.pdf]

XRD Data:

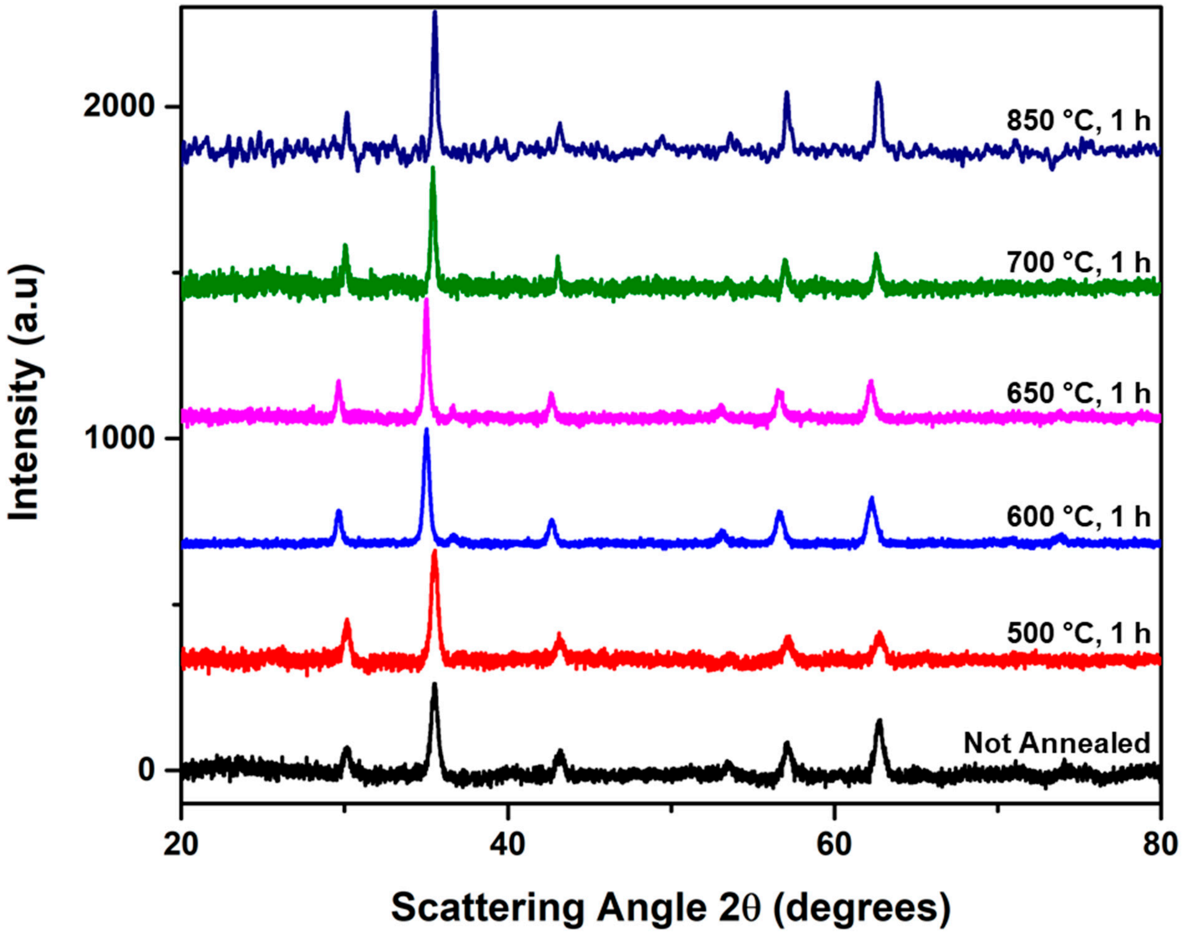

Figure S1. X-ray diffraction (XRD) patterns of 135 nm Fe_3O_4 magnetic nanoparticles (MNPs) and samples annealed for 1 h at 500, 600, 650, 700, and 850 °C.

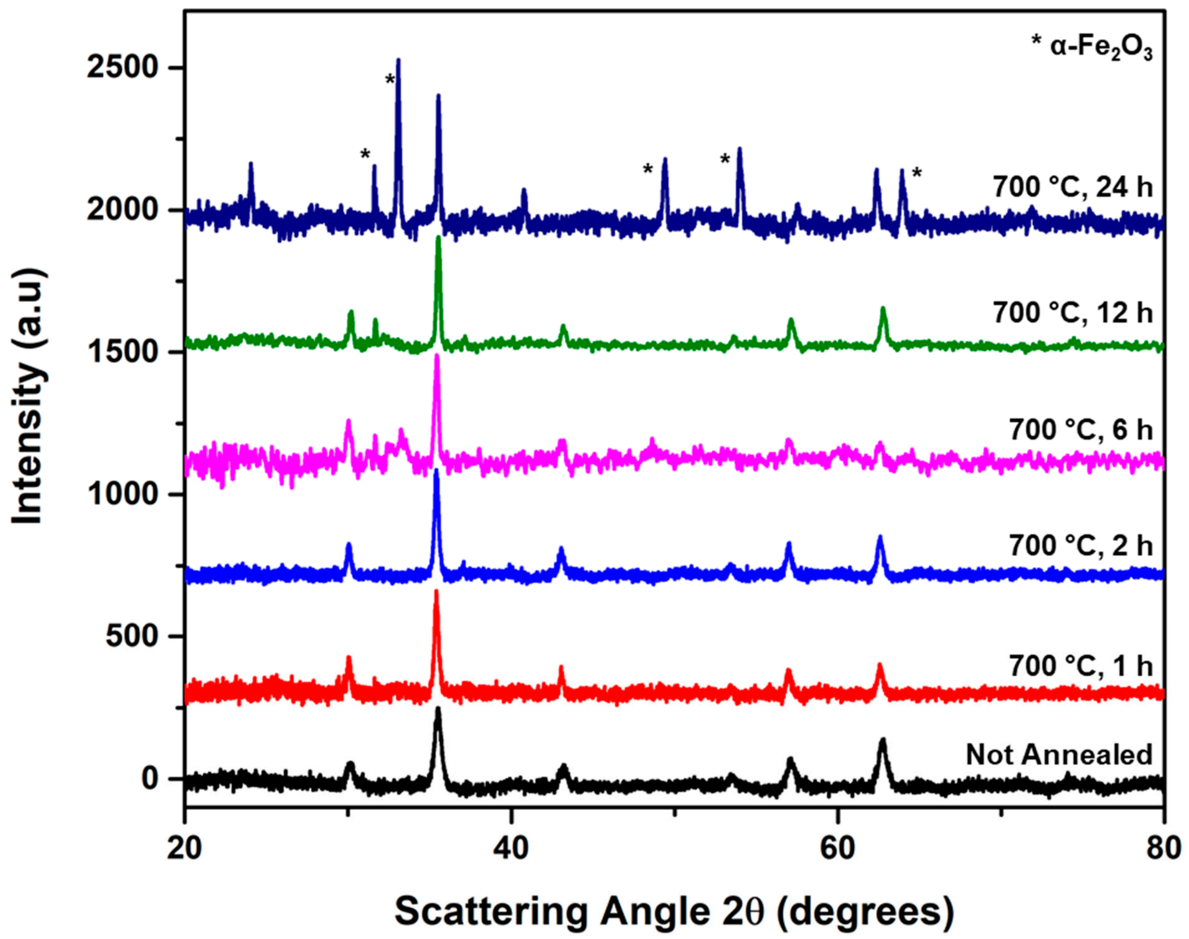

Figure S2. XRD patterns of 135 nm Fe_3O_4 MNPs and samples annealed at 700 °C for 1, 2, 6, 12, and 24 h. Annealing at 700 °C led to oxidation, resulting in the formation of impurities associated with the α-Fe_2O_3 phase.

Table S1. Magnetic properties of 135 nm Fe_3O_4 MNPs and samples annealed at different temperatures for varying durations.

**Sample**	**Original Shape**	**Temp.** (°C)	**Duration** (h)	**Mass** (mg)	***Ms*** (emu/g)	***Ms* Ratio**	***Hc*** (Oe)	***Hc* Ratio**	**Crystallite Size** (nm)	**Mass Error %**[a] (mg)
0	NS[b]	Not annealed	-	5	65.01 ± 1.41	-	52.56 ± 6.95	-	16	0.02
11	NS	500	1	4.1	70.63 ± 1.66	1.1	79.93 ± 7.43	1.5	17	0.02
5	NS	500	2	4.5	73.71 ± 1.62	1.1	95.33 ± 7.52	1.8	17	0.02
6	NS	500	6	4.7	62.68 ± 1.46	1	90.42 ± 7.42	1.7	24	0.02
15	NS	600	1	5.2	75.37 ± 1.52	1.2	98.24 ± 7.40	1.8	23	0.02
14	NS	600	2	6.2	74.81 ± 1.40	1.2	96.85 ± 7.24	1.8	24	0.02
19	NS	650	1	6.20	75.74 ± 1.41	1.2	121.33 ± 7.44	2.3	28	0.02
20	NS	650	2	6.70	76.27 ± 1.37	1.2	105.77 ± 7.25	2.0	28	0.01
12	NS	700	1	4.8	83.92 ± 1.67	1.3	109.77 ± 7.60	2.1	26	0.02
16	NS	700	2	4.6	77.97 ± 1.64	1.2	102.41 ± 7.57	1.9	31	0.02
18	NS	700	6	6.80	84.60 ± 1.42	1.3	112.27 ± 7.28	2.1	35	0.01
22	NS	700	12	1.10	81.56 ± 4.50	1.3	123.63 ± 12.08	2.3	39	0.09
23	NS	700	24	4.60	0.69 ± 0.80	0.02	2039.4 ± 28.63	39	55	0.03
9	NS	850	1	3.7	82.07 ± 1.91	1.3	88.53 ± 7.66	1.7	31	0.03
8	NS	850	2	4.1	77.51 ± 1.74	1.2	91.77 ± 7.58	1.7	-	0.02
2	NS	850	6	6.8	72.72 ± 1.33	1.1	110.83 ± 7.27	2.1	36	0.01
E289	NC[c]	Not annealed	-	9.4	82.75	1.3	120.19	2.3	56	0.01
E289	NC	700	2	4.5	85.81	1.3	90.74	1.7	43	0.02

[a] Scale resolution = 0.1 mg; [b]NS = nanosphere; [c]NC = nanocube

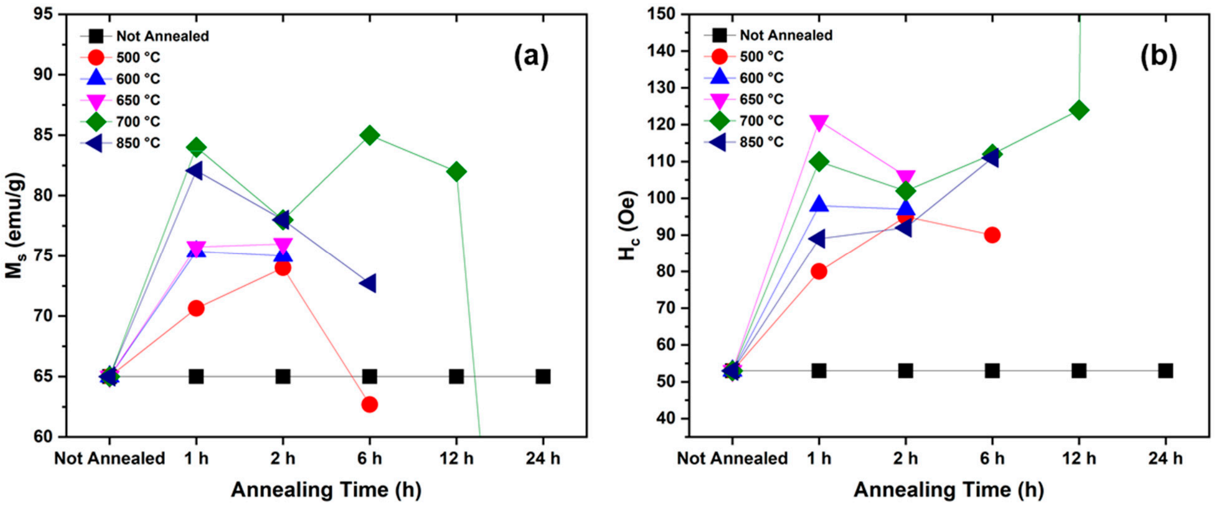

Figure S3. (a) Saturation magnetization (M_s) and (b) coercivity (H_c) of 135 nm Fe_3O_4 MNPs and samples annealed at different temperatures for varying durations.

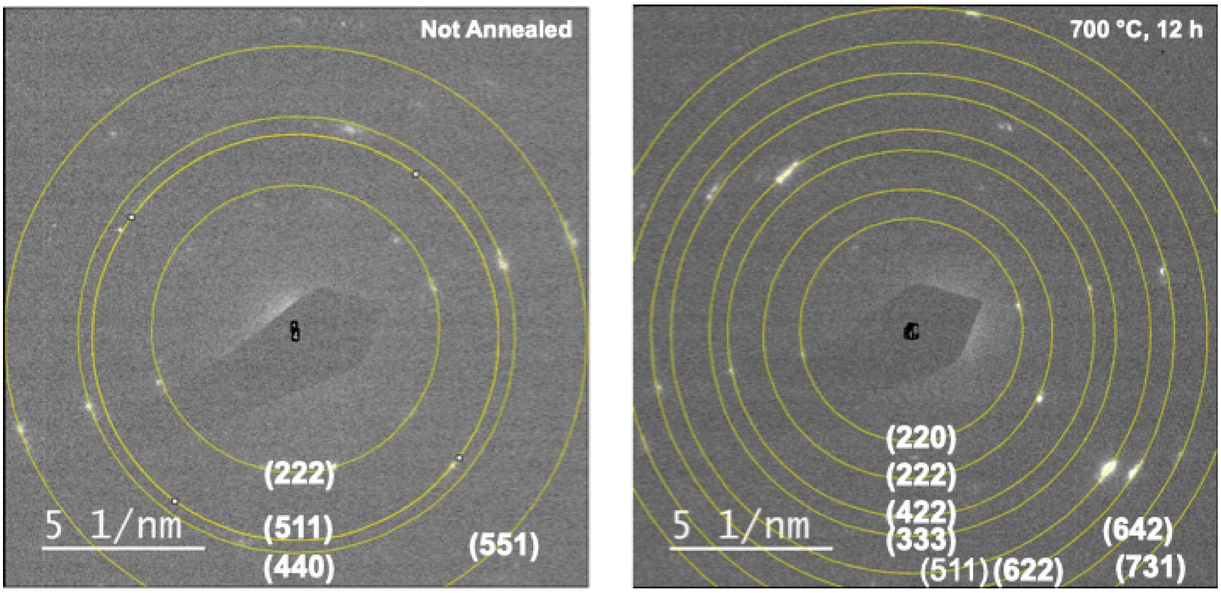

Figure S4. Selected area electron diffraction (SAED) patterns of 135 nm Fe_3O_4 MNPs and samples annealed at 700 °C for 12 h.

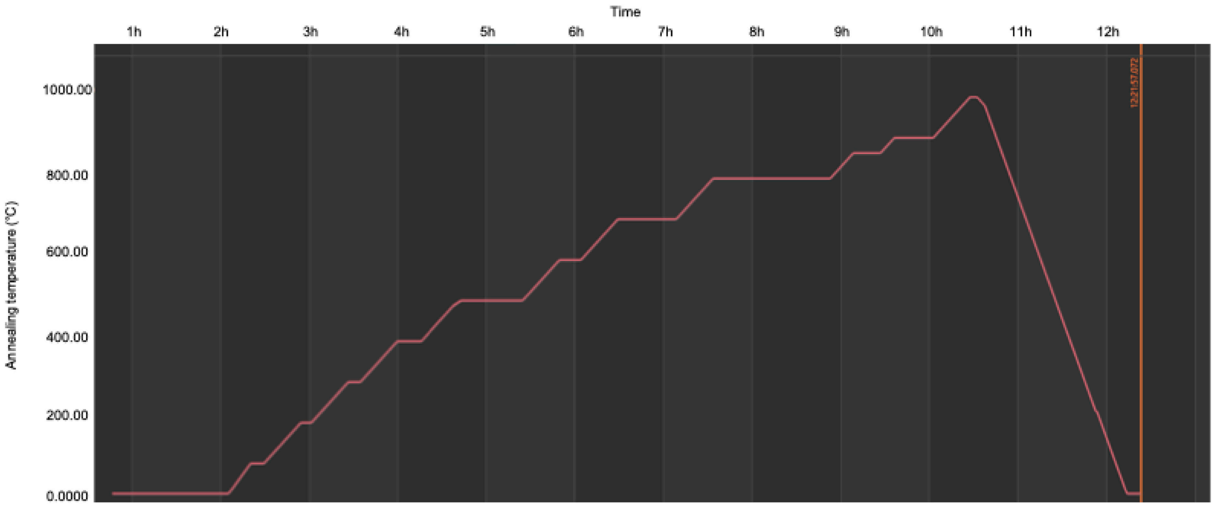

Figure S5. Heating curve for in situ transmission electron microscopy (TEM) measurements.
