# Supplementary figures and images for: Tuning the Crystallite Size, Shape, and Magnetic Properties of Fe3O4 Nanoparticles Using Annealing
Source: Materials (Basel). 2026 Jul 7;19(13):2911. doi: 10.3390/ma19132911 (PMC13362655; doi:10.3390/ma19132911)

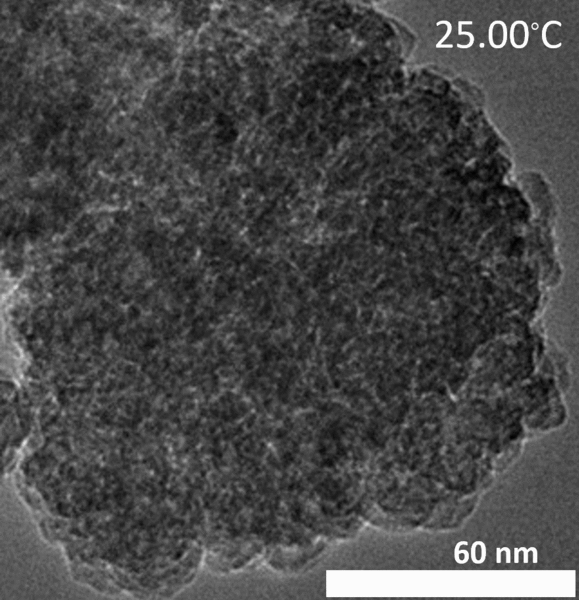

Supplement: Supplementary file 1 [file materials-19-02911-s001.zip › Supplementary Video S1-Fe3O4 in-situ annealing.gif]
